# Efficacy of superficial femoral artery as a recipient in free flap reconstruction around the knee: Four case reports and a literature review

**DOI:** 10.1016/j.jpra.2024.08.001

**Published:** 2024-08-22

**Authors:** Mitsutoshi Ota, Makoto Motomiya, Naoya Watanabe, Kazuya Kitaguchi, Norimasa Iwasaki

**Affiliations:** a Department of Orthopaedic Surgery, Obihiro Kosei hospital Hand Center, Obihiro, Japan; b Department of Orthopaedic Surgery, Faculty of Medicine and Graduate School of Medicine, Hokkaido University, Sapporo, Japan; c Department of Radiological Technology, Obihiro Kosei Hospital, Obihiro, Japan

**Keywords:** End-to-side anastomosis, Free flap, Knee, Soft tissue defect, Superficial femoral artery

## Abstract

**Purpose:**

Reconstructing soft tissue defects around the knee with free flaps presents challenges in recipient vessel selection. Although the superficial femoral artery (SFA) offers exposure ease and anatomical stability, concerns arise regarding its distance from the defect site, difficulty in performing anastomosis and potential peripheral ischaemia. This study aimed to reassess the suitability of SFA as a recipient vessel for knee reconstructions by examining our cases and those from previous reports.

**Methods:**

We reviewed four cases of knee soft tissue defects reconstructed with free flaps using the SFA, detailing surgical techniques and outcomes. Additionally, a comprehensive literature search was conducted for articles on using SFA as a recipient vessel for knee free flaps, using PubMed, Web of Science and EBSCOhost databases.

**Results:**

In all four cases, latissimus dorsi (LD) flaps were used, with end-to-side anastomosis performed using a large slit-shaped arteriotomy. All flaps demonstrated successful survival without complications. Our analysis included 85 cases, comprising four of our cases and 81 cases from 16 articles. Sarcoma resection was the most common aetiology, followed by total knee prosthesis-related defects, trauma and osteomyelitis. Complete flap necrosis occurred in 5% of cases. The LD flap was the predominant choice, alongside other long-pedicle flaps. The SFA provided coverage for all knee areas except the distal lateral patellar region.

**Conclusion:**

Despite the limited evidence, the SFA appears to be a reliable recipient vessel for knee soft tissue reconstruction. Comprehensive understanding of the characteristics of the SFA and flaps used enhances the safety and efficacy of soft tissue defect reconstruction around the knee.

## Introduction

Soft tissue defects around the knee often occur because of post-operative infection following total knee replacement (TKR), severe trauma or tumour resection.^1^^,^^2^ To control wound infection or osteomyelitis and preserve limb function, timely soft tissue reconstruction is essential. Gastrocnemius flaps are typically the first choice, but free flaps are needed for extensive defects or in case of poor local tissue conditions, such as post-trauma and multiple surgeries.^3^ Free flap reconstruction around the knee is challenging owing to anatomical factors such as defect location on the anterior knee, significant joint mobility and risk of vascular compression from knee movement.^2^^,^^4^ Thus, careful preoperative planning, including recipient vessel selection based on defect location and vascular status, is crucial.^1^^,^^5^, ^6^, ^7^, ^8^

Various vessels such as the superior, middle, and inferior genicular arteries, descending genicular artery, descending branch of the lateral circumflex femoral artery, sural artery, anterior and posterior tibial arteries, popliteal artery and superficial femoral artery (SFA) in the adductor canal have been used for free flaps around the knee.^1^^,^^3^^,^^6^ The SFA is stable, provides sufficient blood flow, is easily exposed in the supine position and is less likely to be compressed by knee movement, making it valuable for knee reconstruction.^3^^,^^4^ However, damage to the SFA, the main artery in this region, can impair peripheral blood flow, leading to the inability to use this artery.^9^^,^^10^ Additionally, the SFA is distant from the defect site, has a thick vascular wall making anastomosis challenging and shows significant size discrepancy with flap vessels, thereby, requiring complex end-to-side (ETS) anastomosis. Hence, the choice of using SFA as a recipient vessel varies among surgeons, but it is a necessary option when other vessels are unsuitable owing to trauma-induced spasm or severe scarring.^3^^,^^5^^,^^11^^,^^12^ Reports on using the SFA for free flaps are few, and information on its indications and precautions is insufficient.

This study aimed to examine the detailed conditions of anastomosed vessels and coverage range in four cases of knee defects reconstructed with free flaps using the SFA. Additionally, we reviewed past reports and re-evaluated the indications for using the SFA in knee soft tissue reconstruction.

## Patients and Methods

**Figure 1:**
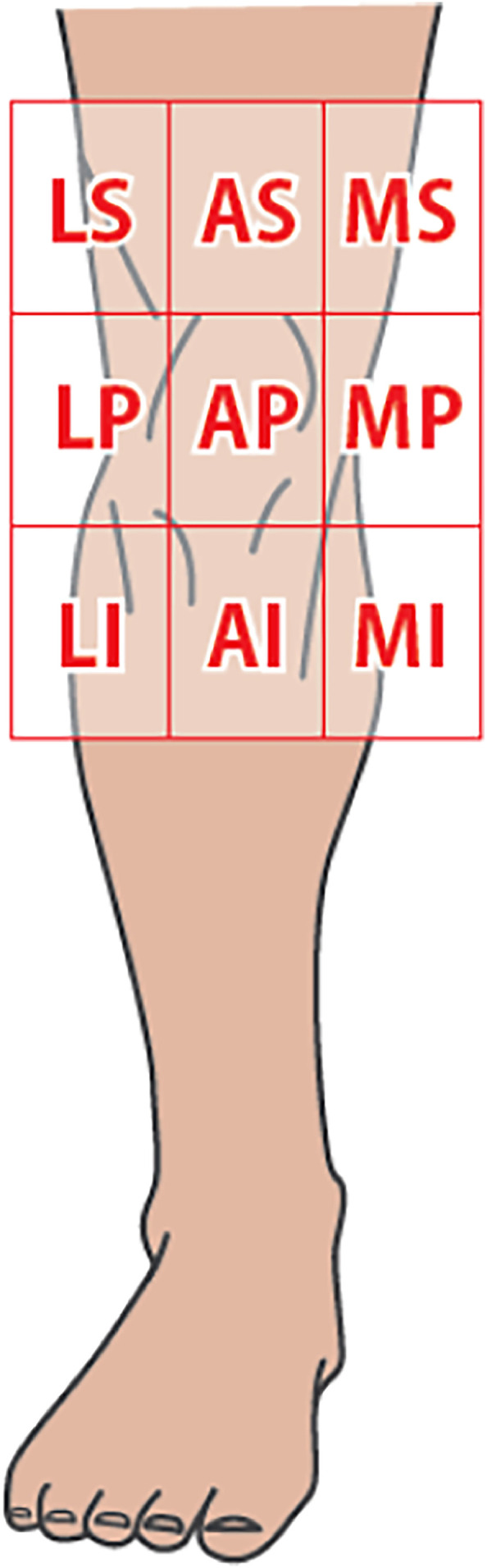
Classification reported by Yuen et al.^12^ of soft tissue defects around the knee. The knee region is divided into 9 zones centred around the patella, classified horizontally into 3 regions (M: medial, A: anterior and L: lateral) and vertically into 3 regions (S: suprapatellar region, P: patellar and parapatellar region and I: infrapatellar region).

This study, approved by our institutional ethics committee (approval number: 2024-01), analysed four cases of free tissue transfer using the SFA as the recipient vessel for knee soft tissue defect reconstruction conducted in our department from August 2019 to December 2022. We examined the patients’ backgrounds, aetiology, flap types, defect locations, flap sizes, outcomes and complications. Intraoperative vessel measurements were made by saving images with a scale under the microscope and measuring post-operatively. Vessel size discrepancy was calculated by dividing recipient diameter by flap pedicle diameter, and the expansion rate by dividing arteriotomy length by flap pedicle diameter. Soft tissue defect locations were categorised into nine regions based on Yuen et al.’s classification,^12^ dividing the knee area centred on the patella into three transverse (medial, anterior and lateral) and three longitudinal regions (suprapatellar, patellar/parapatellar and infrapatellar) (Figure 1).

### Surgical Techniques

**Figure 2:**
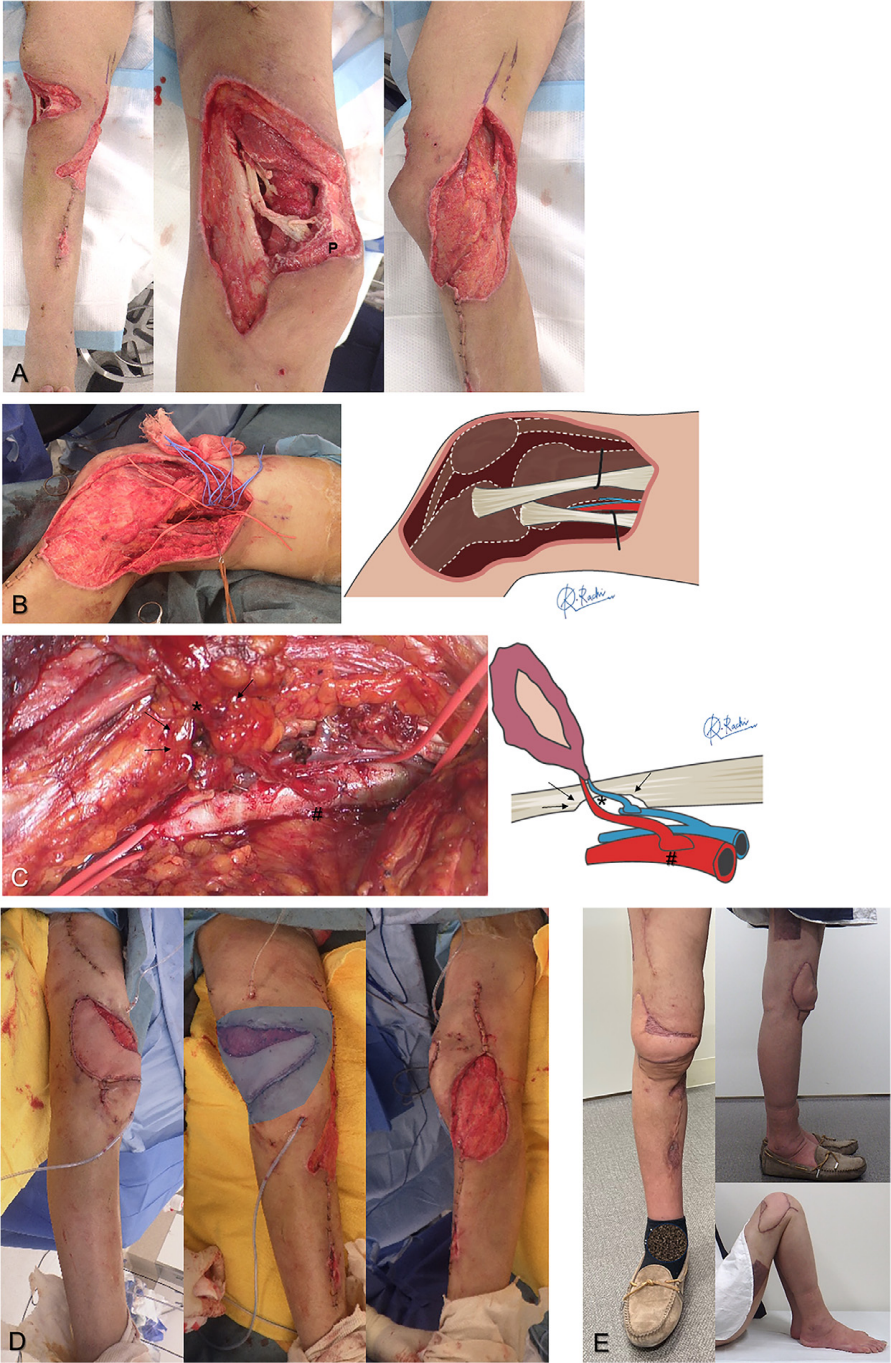
A 61-year-old female with an open injury to the right knee joint due to a traffic accident (Case 1). **A**: After debridement, extensive soft tissue defects with joint capsule disruption was found around the knee (P: patella). **B**: Reconstruction of the soft tissue defect was performed using a free latissimus dorsi (LD) flap (skin paddle: 16 cm × 5 cm, muscle flap: 23 cm × 13 cm) 21 days after the injury. The superficial femoral artery (SFA) was dissected between the sartorius and adductor muscles and used as the recipient vessel. **C**: Concerns about potential kinking of the vascular pedicle (asterisk) due to the deep anastomosis site (hashmark) to the SFA led to a portion of the sartorius muscle being excised (arrow) to relieve pressure on the vascular pedicle. **D**: The LD flap was spread out, and muscle was laid into the pocket-like defect area (shaded area), covering the extensive three-dimensional defect. **E**: No post-operative complications occurred, and split-thickness skin grafting was performed 6 days after the flap surgery. Appearance at one and a half years post-operatively showing complete engraftment of the flap and skin graft with preservation of a good range of motion. The patient returned to her former job as a skating instructor without any complaints.

**Figure 3:**
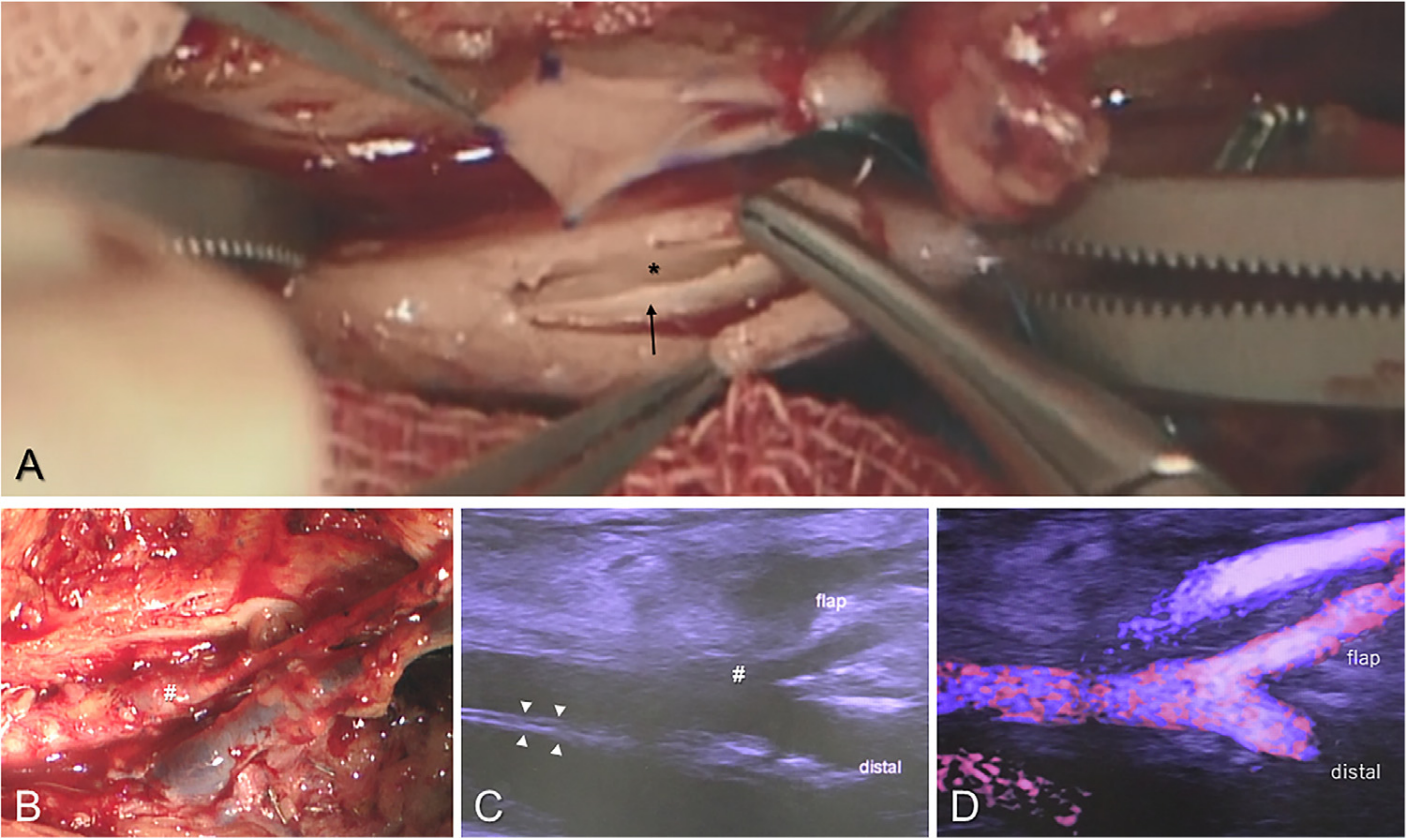
Arterial anastomosis to the atherosclerotic SFA (case 2). **A**: Clear visualisation of the denuded intima (black arrow) and true lumen of the vessel (asterisk) through a large window. By directing the needle away from the vessel lumen, precise suturing can be achieved without stripping the vessel's intima. **B**: Post-anastomosis appearance showing the bulging anastomosis site (hashmark) over the thick SFA. **C**: Ultrasound image at 3 weeks after flap surgery revealing a well-expanded window on the thick arterial wall (arrowhead). **D**: Colour Doppler showing no turbulence at the anastomosis site, ensuring good blood flow to the flap and recipient periphery.

Preoperatively, we confirmed the absence of significant calcification around the SFA in the adductor hiatus using X-ray. With the hip in maximal external rotation and the knee flexed, the SFA was dissected between the sartorius and adductor muscles (Figure 2B). The flap pedicle was harvested to its maximum length. All vascular anastomoses were performed using microscopic parachute end-to-side techniques.^13^^,^^14^ Briefly, the flap vessel was significantly widened, and a large slit-shaped window was created in the recipient vessel. Using the parachute technique, anastomosis was first performed at the heel, a high-risk site for blood leakage, followed by continuous suturing to the posterior and anterior walls to control bleeding. When the vessel wall was thickened due to arteriosclerosis, we adjusted the suturing direction to avoid intimal detachment (Figure 3A). For the recipient vein, a large branch of the superficial femoral vein was used. When transplanting the flap to the anterior or lateral knee defects, the pedicles sometimes became constricted in areas where they crossed the sartorius fascia, necessitating partial resection of the sartorius muscle to prevent vascular pedicle kinking (Figure 2C).^4^

### Ultrasonographic Vascular and Blood Flow Measurement

Approximately 3 weeks post-operatively, blood flow measurements were performed using Doppler ultrasound by a specialist. Following previous study conditions, patients lay supine and rested until their blood pressure stabilised.^14^ Using a high-resolution ultrasound device (Aplio 1800, Canon Medical Systems) with a 12-18 MHz linear probe, we measured the inner diameter and blood flow of vessels at two locations: proximal to the anastomosis site in the recipient artery and distal to the anastomosis in the flap artery. The transducer angle was kept <60° during measurements, taken three times each, and averaged. Additionally, colour Doppler mode was used to investigate the turbulence at the anastomosis site (Figure 3D).

### Literature Search Method

Following the PRISMA 2020 guidelines, a comprehensive literature search was conducted on January 10, 2024, using PubMed, Web of Science and EBSCOhost using the terms ‘free flap’ AND ‘superficial femoral artery’ or ‘free flap’ AND ‘knee’ to maximise relevant case report retrieval, including all literature published up to that date.^15^

### Study Selection Criteria

**Figure 4:**
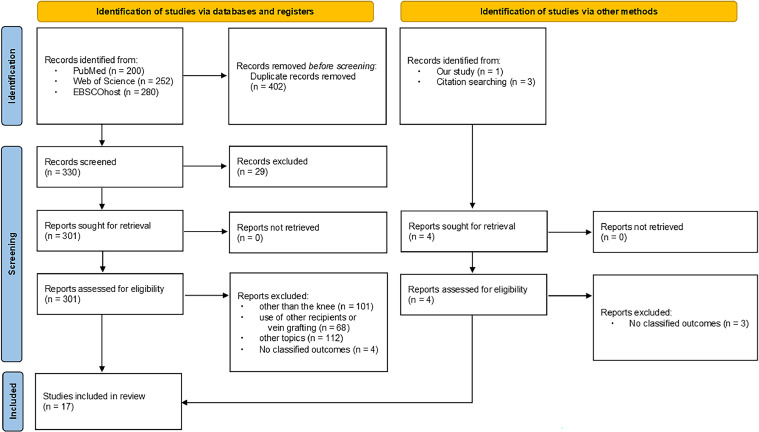
PRISMA 2020 flow diagram.

The PRISMA 2020 flow diagram illustrates the study selection process (Figure 4). Articles were screened based on predefined inclusion and exclusion criteria. Inclusion criteria encompassed case series of free flaps around the knee using the SFA as the recipient vessel. Exclusion criteria included non-English articles, studies on animals or cadavers, review articles without specific case examples, studies on free tissue transfer for amputation stump reconstruction, studies unrelated to the theme, articles with unclear records on recipient vessels, studies using vein grafts for recipient vessel anastomosis and articles without flap outcome reports.

### Data Extraction and Synthesis

Two independent reviewers initially screened the titles and abstracts to assess the eligibility. Full texts of potentially eligible studies were collected, and data on study characteristics (author, publication year and journal), patient background, aetiology, flap type, flap complications, anastomosis techniques and defect location were extracted. Data analysis was performed using only the available data. Case reports were qualitatively assessed using the National Institutes of Health evaluation tool.^16^ Discrepancies were resolved by consensus or consultation with a third reviewer.

## Results

### Case Profiles and Flap Characteristics

Background data for all patients and flaps are shown in Table 1. Among the knee soft tissue defect cases, one was trauma-related and three were infection-related. All cases had joint capsule disruption and cavity exposure following multiple debridements. Soft tissue reconstruction was performed using a free latissimus dorsi (LD) myocutaneous flap with the SFA as the recipient vessel. Defects were located outside the lateral and infrapatellar regions. For anterior, patellar and parapatellar reconstructions, the joint capsule was sutured with the LD fascia, and the skin was sutured to the flap for layered repair. All flaps survived without complications. One trauma case required secondary split-thickness skin grafting. One case related to TKR required joint arthrodesis, but limb salvage and independent ambulation were achieved in all cases.

**Table 1:** Case profiles and flap characteristics.

Case no.	Age (yr)	Sex	Comorbidity	Aetiology	Debridement before flap	Flap type	Location⁎	Fap size			Complication	Additional surgery
								Muscle (cm^2^)	Skin paddle (cm^2^)	Pedicle length (cm)		
1	61	F	None	Trauma	9	LD	AP, LS, LP	23 × 13	16 × 5	9.0	None	Skin graft
2	48	F	DM, Blount's disease	Osteomyelitis of Tibia (tibial osteotomy in childhood)	4	LD	AS, AP, AI	18 × 15	13 × 8	6.0	None	Bone graft
3	69	F	RA	TKR-related defect	3	LD	AS, AP, AI	17 × 13	17 × 8	8.5	None	Knee joint arthrodesis
4	49	M	None	Osteomyelitis of Tibia (knee ligament injury/popliteal artery injury in adolescents)	4	LD	MP, MI, AP	17 × 7	17 × 7	8.0	None	None

DM: diabetes mellitus, LD: latissimus dorsi myocutaneous flap, RA: rheumatoid arthritis, TKR: total knee replacement

⁎ : according to Yuen's classification^12^

### Vascular Measurements of Recipient and Flap Pedicles

Data on vascular anastomoses are shown in Table 2. The outer diameter of SFA ranged from 4.5 to 6.3 mm, whereas the flap pedicle diameter ranged from 1.5 to 2.2 mm, resulting in a size discrepancy of 2.0 to 4.2 times. The arteriotomy length was 5.5 to 8.0 mm, averaging 3.7 times the flap pedicle diameter. Extensive intimal detachment due to arteriosclerosis was observed in two cases, necessitating the adjustment of the anastomosis direction. In one case, minor blood leakage was controlled using an additional suture. At three weeks post-operatively, ultrasound showed no turbulence at the anastomosis site. The internal diameter of SFA ranged from 3.4 to 5.1 mm, whereas the flap pedicle diameter ranged from 0.9 to 1.9 mm, with a size discrepancy of 1.8 to 5.3 times. All SFAs had high-volume blood flow >90 ml/min at the anastomosis site.

**Table 2:** Vascular measurements of recipient and flap pedicles.

Case no.	Intraoperative measurements					Post-operative ultrasound measurement				
	Recipient	Flap pedicle	Length of arteriotomy (mm)	Vessel size discrepancy	Expansion rate of flap pedicle	Recipient		Flap pedicle		Vessel size discrepancy
	OD (mm)	OD (mm)				ID (mm)	Flow volume (ml/min)	ID (mm)	Flow volume (ml/min)	
1	6.3	1.5	6.0	4.2	4.0	5.1	98	1.8	20	2.8
2	4.5	1.5	8.0	3.0	5.3	3.5	101	1.1	26	3.2
3	5.0	2.5	7.0	2.0	2.8	3.4	123	1.9	30	1.8
4	6.0	2.2	5.5	2.7	2.5	4.8	219	0.9	22	5.3
Average	5.5	1.9	6.6	3.0	3.7	4.2	135	1.4	25	3.3

OD: outer diameter, ID: inner diameter

### Review Results

No comparative studies on using the SFA as the recipient vessel were found; therefore, we focused on case series and reports. We reviewed 16 articles alongside our cases,^2^^,^^4^^,^^7^^,^^12^^,^^17^, ^18^, ^19^, ^20^, ^21^, ^22^, ^23^, ^24^, ^25^, ^26^, ^27^, ^28^ totalling 85 cases with identifiable outcomes (Table 3). Among the case series with three or more SFA recipient cases, only three studies exclusively used the SFA,^2^^,^^4^^,^^23^ whereas the others selected vessels case-by-case. The most common cause of defects was sarcoma resection (47%, 37/78 cases), followed by TKR-related infection or defect (21%, 16/78), trauma (15%, 12/78), osteomyelitis or skin necrosis (13%, 10/78) and knee joint contracture (4%, 3/78). Myocutaneous flaps (LD or rectus abdominis) were the most frequently used (85%, 66/78), followed by fasciocutaneous flaps (anterolateral thigh flap (ALT) or fascia lata flap) (15%, 12/78). Complications included complete flap necrosis (5%, 4/85) and partial necrosis (6%, 5/85). No cases of peripheral ischaemia following ETS to the SFA were reported.

**Table 3:** Summary of previous reports on free flaps using the superficial femoral artery as the recipient vessel.

Author	Year	Number of flaps	Aetiology	Flap type	Flap-related complications						Quality assessment score
					Arterial thrombosis	Venous thrombosis	Takebacks	Complete failure	Partial failure	Others	
Fisher et al.^4^	1983	3^#^	Osteomyelitis	LD	0	0	0	0	0		7/9
Yuen et al.^12^	1996	5	Trauma, soft tissue necrosis	LD	ND	ND	ND	1	0		6/9
Hallock^20^	1997	1	Trauma	LD + SA + Scapula	0	0	0	0	0		7/9
Manoso et al.^23^	2006	11^#^	Sarcoma resection	LD, RA	0	ND	ND	0	0	1 venous congestion	6/9
Peters et al.^24^	2008	2	TKR-related defect, contracture	LD, ALT	1	0	1	2	0	1 hematoma	7/9
Hierner et al.^2^	2009	14^#^	TKR-related defect	LD	1	1	2	0	1	5 skin breakdowns	7/9
Dragu et al.^19^	2008	1	Osteomyelitis	LD	0	0	0	0	0	1 hematoma	6/9
Popov et al.^26^	2010	15*	Sarcoma resection	LD, LD + bone, ALT, FL	ND	ND	ND	1	0	3 minor wound complication	6/9
Louer et al.^7^	2015	7^$^	Trauma, sarcoma resection, osteomyelitis	almost LD	1	0	ND	0	0		5/9
Struckmann et al.^28^	2017	1	Trauma	ALT	0	0	0	0	0		7/9
Bigdeli et al.^17^	2018	8	Trauma	LD + parascapular	0	0	ND	0	3		6/9
Khoshnevis et al.^21^	2018	1	Contracture	LD	0	1	1	0	0		7/9
Philandrianols et al.^25^	2018	3	Sarcoma resection	ALT	0	0	0	0	0		6/9
Sapino et al.^27^	2019	2	Sarcoma resection	ALT + VL + FL	0	0	ND	0	1	1 venous congestion	6/9
Lara et al.^22^	2021	1	Contracture	ALT	ND	ND	1	0	0	1 pedicle thrombosis	6/9
Cohen et al.^18^	2023	6	Sarcoma resection	LD, RA	0	0	0	0	0		5/9
***Our study***		***4^#^***	***Trauma, TKR-related defect, osteomyelitis***	***LD***	***0***	***0***	***0***	***0***	***0***		***7/9***

Total		85			3 (5%)	2 (4%)	5 (14%)	4 (5%)	5 (6%)		

TKR: total knee replacement, LD: latissimus dorsi myocutaneous flap, SA: serratus anterior flap, RA: rectus abdominis flap, ALT: anterolateral thigh flap, VL: vastus lateralis flap, FL: tensor fascia lata flap, #: SFA used in all cases, *: either SFA or popliteal artery used as recipient, $: Detailed aetiology and type of skin flap were excluded from consideration in this study due to ambiguity.

**Figure 5:**
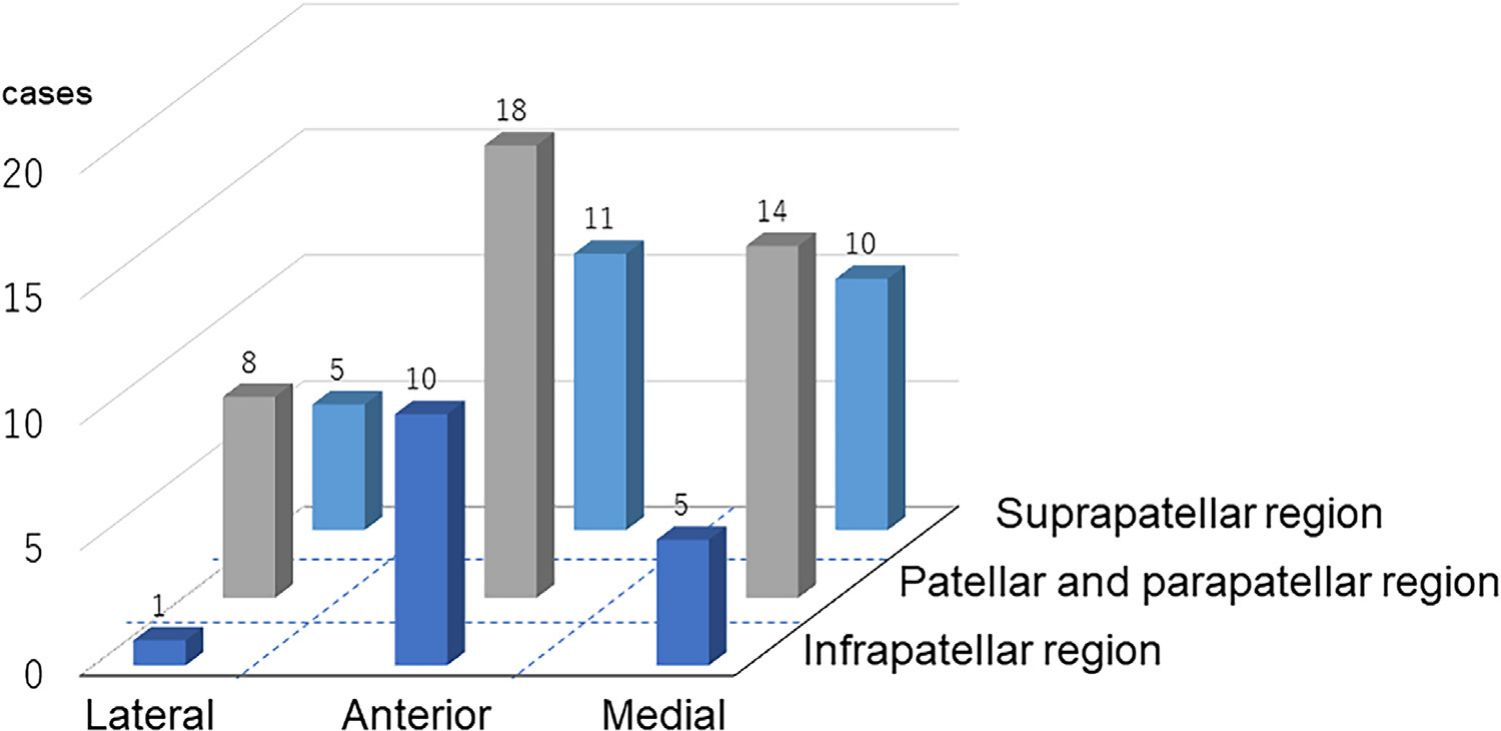
Summary of the coverage areas of cases reported in previous reports.

All cases used the ETS technique, with detailed ETS descriptions provided only by Khoshnevis et al.,^21^ who used an arteriotomy size three times the flap pedicle diameter. Coverage areas for cases without necrosis included 17 LD flaps, two LD combined with scapula flaps, four ALT flaps, and one ALT combined with vastus lateralis and fascia lata flap (Figure 5). The patellar and parapatellar regions were the most common coverage areas. Only one case included the lateral and infrapatellar regions; all other regions had five or more successful coverage cases.

## Discussion

In this study, we investigated the reconstruction of soft tissue defects around the knee using free flaps with the SFA as the recipient vessel. We demonstrated for the first time, the characteristics of the SFA as a recipient vessel for free flaps, including its large diameter, thick vascular wall and high flow, as well as the significant size difference between the flap pedicle and SFA. Using a free LD flap with the SFA for extensive knee soft tissue defects resulted in no flap complications or peripheral ischaemia. Haddock et al.^9^ described ideal recipient vessel characteristics, including adequate diameter, consistent anatomy, ease of exposure, ease of patient positioning, protected location from injury and lack of morbidity from use. The SFA is considered to possess several of these properties. However, several reports advise against using the SFA as a recipient vessel,^6^^,^^10^ and even among reports that used SFA, there were relatively few advocating for its proactive use.^2^^,^^4^^,^^23^ Past reports have pointed out the challenges of free flap reconstruction around the knee,^2^^,^^4^ but understanding the detailed characteristics of the SFA and coverage range of appropriate flaps reaffirms that SFA is a valuable option for knee reconstruction.

**Figure 6:**
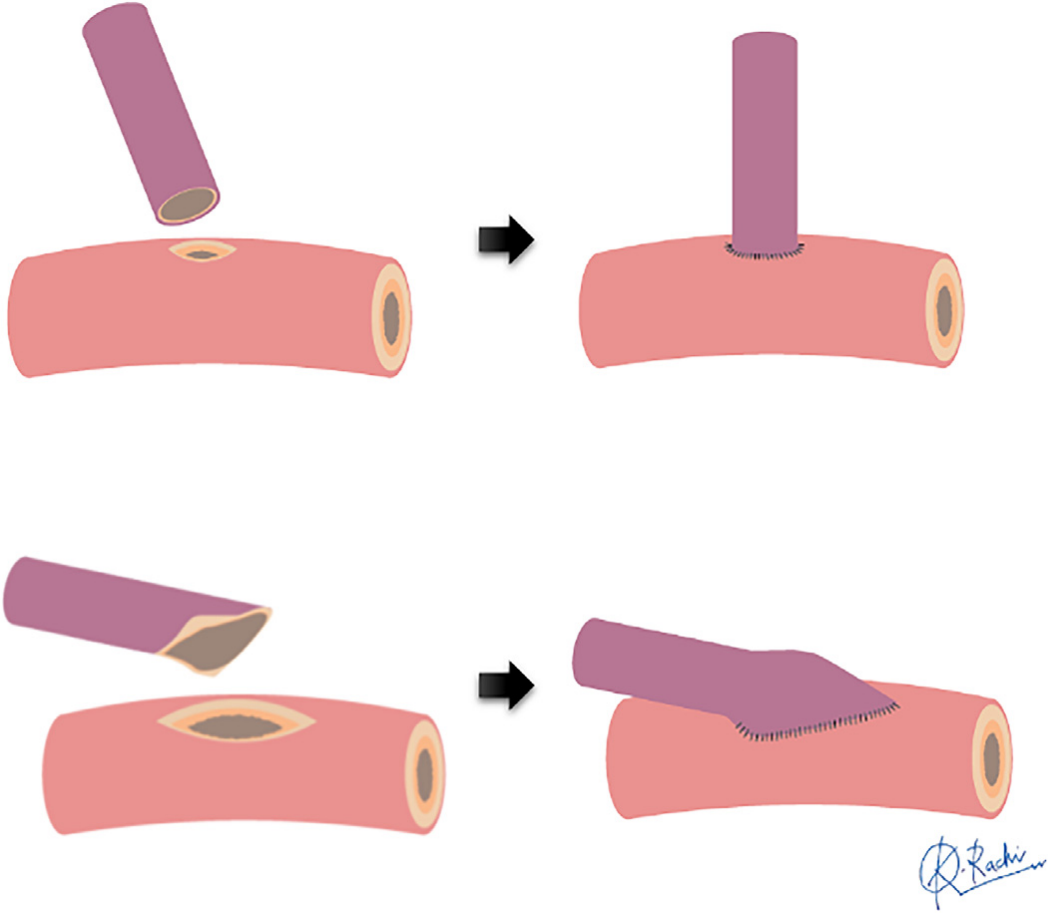
Schematic of two types of end-to-side anastomosis.

In major lower limb arteries such as the SFA, arterial sclerotic changes due to severe peripheral vessel disease and vascular fibrosis from trauma or infection, can pose challenges for anastomosis.^10^^,^^24^^,^^29^ Given the distinct features of recipient vessels, it is crucial to have information regarding vascular anastomosis techniques. However, according to the results of this review, only one study made specific mention of ETS methods.^21^ Generally, the arteriotomy to the recipient vessel is made the same size as the flap pedicle diameter in ETS (Figure 6).^30^^,^^31^ However, ensuring a secure grasp of the thick-walled SFA lumen and capturing the easily detached intima for anastomosis can be challenging, especially from a small opening. Additionally, in cases of calcified arteries, performing an arteriotomy without damaging the intima can be difficult, and reports suggest that a large slit-shaped arteriotomy is more suitable.^32^ Khoshnevis et al.^21^ discussed the utility of large window ETS, which involves an arteriotomy that is approximately three times the size of the flap pedicle diameter, and our cases also had arteriotomies of nearly the same size, averaging 3.7 times. The ETS with slit-shaped large arteriotomy allows for minimal intimal damage during arteriotomy, enabling secure needle passage while ensuring a clear view of the intima. It is considered a useful distal anastomosis method for free flaps with the SFA as the recipient vessel.^13^^,^^14^^,^^33^

Regarding flap survival, total flap necrosis of free flaps with the SFA as the recipient vessel around the knee was 5%, a rate comparable to previous reports on free flaps in the extremities.^34^^,^^35^ Using small arteries around the knee can lead to vasospasm in the recipient vessel owing to damage to the surrounding tissue,^1^^,^^5^ whereas free flaps with SFA as the recipient vessel did not result in complications related to refractory vasospasm, including those in our cases. Complications related to arterial occlusion of the flap were observed in 4% (3/85) of cases in past reports, but no cases were reported of peripheral ischaemia due to recipient stenosis or steal phenomenon at the anastomosis site. ETS to the main artery has been reported to have no long-term effects,^36^ and it is considered safe to use the SFA as the recipient vessel with appropriate anastomosis.

Regarding flap types, according to past reports, as the SFA is located away from the knee, flaps with long vascular pedicles such as the LD, rectus abdominis, or ALT were frequently used. Additionally, myocutaneous flaps were the most commonly used flap type, accounting for 85% of reported cases. Using myocutaneous flaps for soft tissue defect reconstruction around the knee offers several advantages.^2^^,^^4^^,^^37^ It is possible to extend the pedicle length by incorporating the proximal part of the muscle body into the vascular pedicle. Myocutaneous flaps have elasticity, allowing the transplanted tissue to stretch and contract with the knee motion. They can fill the three-dimensional defect around the knee and the deep part of the flap can function as a pseudo-capsule around the knee. Additionally, they are rich in muscle tissue with abundant blood flow, which is useful in controlling infection. All our cases involved soft tissue defects with exposed joints, except for one case where arthrodesis was necessary, knee joint function could be maintained by repairing the joint capsule and skin layer-by-layer using the LD flap.

Regarding flap coverage range, past reports have shown that using free flaps with the SFA as the recipient vessel enables wide range of defect coverage around the knee except for the lateral and infrapatellar regions. However, partial necrosis was observed in 6% of cases. The LD flap, which is the most commonly used, has a Mathes and Nahai type V vascular supply,^3^ and because the distal portion receives blood supply from nutrient vessels other than the thoracodorsal artery, it is important to be aware that the LD flap is prone to peripheral necrosis. In cases requiring coverage of the distal to anterior and/or lateral and infrapatellar regions, consideration should be given to changing the recipient vessel to the saphenous or popliteal artery.^8^^,^^12^^,^^38^

Concerning the indications for using the SFA as the recipient vessel, various aetiologies of cases have been adapted in past reports. Based on the results of this study, the SFA is considered useful as the recipient vessel in the following cases: cases with extensive soft tissue defects after sarcoma resection, cases requiring reconstruction of the anterior midline of the knee (AS/AP/AI) due to revision surgery for TKR, cases with severe trauma or osteomyelitis resulting in damage to the surrounding soft tissue defect and unreliable small arteries around the knee as recipient vessels and cases of knee contracture with difficulty in exposing the recipient vessels within scar tissue. There are several potential recipient vessels around the knee, but it is essential to be prepared to switch intraoperatively to the reliable SFA if the initially planned small recipient vessels cannot be used.

When performing free flaps using the SFA as the recipient vessel, it is necessary to evaluate the SFA for the presence of vascular lesions such as calcification or occlusion beforehand.^39^ In cases of highly calcified lesions in the SFA, it is desirable to explore other recipient vessels, but if it is necessary to use a calcified vessel as the recipient, special techniques using artificial vessels or vein grafts should be prepared.^5^^,^^29^^,^^40^

Limitations of this study include the small number of cases and the lack of proper statistical analysis. As this study was conducted in a single centre with a small patient cohort, it is difficult to extrapolate the findings to other populations. This limitation means that caution is required when comparing our results with those of other studies or different populations. The specific circumstances and patient characteristics at our institution may not be representative of other settings, which further limits the generalizability of our findings. In the future, larger multi-centre studies are needed to validate our results and allow for proper statistical analysis. Such studies would help in generalising the findings on the use of the SFA as the recipient vessel in a broader context. Despite these limitations, our study provides valuable insights into the treatment of high-risk patients. We believe that our findings can contribute to the development of new approaches and stimulate further research in this area.

## Conclusions

In this report, we investigated four cases of soft tissue defects around the knee reconstructed using free flaps with the SFA as the recipient vessel, examined the conditions of the anastomotic vessels and coverage range of the soft tissue defects and reconfirmed the indications for using the SFA as the recipient vessel based on past reports. From this study, it is evident that by transplanting myocutaneous flaps with long vascular pedicles into the SFA as recipients, soft tissue defects can be reliably covered up to the region excluding the lateral distal area of the knee. Recognising the characteristics of the SFA, including its large diameter, thick vascular wall and high flow, and acquiring proficiency in ETS techniques capable of addressing arteriosclerosis and calcification are essential. Understanding the features of the SFA, free flaps with SFA as the recipient vessel can become a more valuable option for reconstructing extensive soft tissue defects around the knee.

## Conflicts of interest

None.
